# Two pathogens, one disease: Rethinking leprosy diversification through ancient and modern genomes

**DOI:** 10.1002/ctm2.70631

**Published:** 2026-02-20

**Authors:** Maria Lopopolo, Charlotte Avanzi, Nicolás Rascovan

**Affiliations:** ^1^ Institut Pasteur Université Paris Cité Evolutionary Dynamics of Infectious Diseases Unit Paris France; ^2^ Institut Pasteur Université Paris Cité, CNRS UMR 2000 Microbial Paleogenomics Unit Paris France; ^3^ One Health Institute University of Zürich Zürich Switzerland; ^4^ Institute of Evolutionary Medicine University of Zürich Zürich Switzerland

1

Leprosy is one of humanity's oldest infectious diseases and has historically been attributed to *Mycobacterium leprae*, the canonical agent described in medical texts and still responsible for most diagnosed cases worldwide. However, just over a decade ago, a second leprosy‐causing species, *Mycobacterium lepromatosis*, was discovered, challenging the long‐standing assumption that a single bacterial species underpinned the full clinical spectrum of Hansen disease.^1^ Early reports suggested that *M. lepromatosis* was rare and geographically restricted, and its role or relevance in human disease was initially met with scepticism. Yet, it has since been reported in more than one hundred human cases, predominantly in the Americas. Paradoxically, despite this growing clinical footprint, genomic data have remained extremely limited, consisting until recently of only three strains from Mexican patients and seven genomes from infected red squirrels in Ireland and Great Britain, leaving major questions about its current and past diversity, and the origins of the species. By contrast, ancient and modern DNA work on *M. leprae* ^2^, ^3^ has demonstrated how integrating extant and time‐calibrated genomes can reconstruct pathogen origins, diversification, and historical spread, all aspects still unresolved for *M. lepromatosis*.

To address this gap, the geographic extent and evolutionary diversity of *M. lepromatosis* were investigated using a strategy built around the region where most cases have been reported: the Americas.^4^ We assembled and analysed a continent‐wide collection of contemporary biopsies and tissues from humans suspected of leprosy, aiming to obtain genome‐wide data that could resolve population structure and diversity, divergence times, and lineage turnover. However, modern sampling alone could not determine when or where the species became established. We therefore screened archaeological human remains predating European contact, reasoning that ancient infections would provide definitive evidence of long‐standing endemicity and directly inform competing hypotheses about the emergence of leprosy, as a disease, in the Americas. This combined approach yielded the detection of *M. lepromatosis* in 34 contemporary samples by species‐specific quantitative polymerase chain reaction (qPCR) and in three ancient individuals, with genome‐wide data recovered from 23 modern genomes and three ancient genomes through a combination of high‐throughput sequencing and targeted in‐solution enrichment. To strengthen downstream genotyping and phylogenetic inference, we also generated an improved reference genome (NHDP‐LPM‐385) using long‐ and short‐read sequencing, enabling higher‐confidence reconstruction of fine‐scale relationships among closely related strains. Strikingly, the recovered ancient genomes spanned from North and South America, demonstrating that *M. lepromatosis* infected human populations at a continent‐wide scale in the Americas long before European contact. These data establish that leprosy in the continent cannot be understood solely through the post‐contact introduction of *M. leprae*.

From a biomedical standpoint, the most immediate implication of these findings is that *M. lepromatosis* is not a recent or geographically marginal agent, but a deeply diversified pathogen whose lineages reflect long‐term persistence and dispersal across the Americas. This matters because deep genomic divergence, within *M. lepromatosis* and between *M. lepromatosis* and *M. leprae*, can plausibly translate into phenotypic differences relevant to clinical care, including variation in virulence, immune recognition, host range, and possibly response to treatment. Our expanded genome set allowed re‐estimating the divergence time between *M. leprae* and *M. lepromatosis* to ∼0.7–2 million years ago, far more recent than earlier estimates^5^, ^6^ but still predating both the settlement of the Americas and the origin of *Homo sapiens* itself, consistent with a long evolutionary history independent of modern humans’ demography. At the species level, multiple principal *M. lepromatosis* lineages are now recognised, including a deeply branching modern lineage represented by the NHDP‐LPM‐9 strain (and supported by NHDP‐LPM‐6). This lineage is basal to all others and exhibits thousands of single‐nucleotide variants relative to the reference, suggesting an independent diversification history–and likely a substantially unsampled related diversity– in yet uncharacterized human or animal host populations. Notably, NHDP‐LPM‐9 also shows evidence of an accelerated substitution rate consistent with a hypermutator phenotype, likely associated with missense mutations in several DNA repair genes (including *mfd*, *dnaE*, *uvrD2*, *recA*, *ruvA*, *ruvB* and *RNaseH1*), but without the classic drug‐resistance signatures described in *M. leprae* hypermutators.

Our findings raise practical questions: if *M. lepromatosis* has circulated for millennia throughout the Americas, why does it still appear rare in routine clinical reporting? A parsimonious explanation is that rarity is partly observational: most diagnostic workflows have historically focused on *M. leprae*, and *M. lepromatosis* testing is uneven across regions, creating geographic and clinical blind spots. In parallel, the documented involvement of wildlife, including the striking signal from red squirrels in the British Isles,^7^ strongly motivates a One Health framing, where zoonotic maintenance could buffer the pathogen against human‐focused control measures and contribute to sporadic re‐emergence. Finally, limited sequence data from outside the Americas remains a critical bottleneck. For example, despite a very few clinical cases reported in Asia, the only available genetic information from this continents comes from the Singapore strain Sg‐1 (based on PCR fragments) from which the *mmaA3* locus shares a single informative SNP with the recently emerged, predominant clade in North America (Figure 1), a tantalizing but clearly insufficient hint that some Asian infections might originate from recently diverged lineages in the Americas. The clinical and translational priority is therefore clear: expand genomics beyond the Americas, while building scalable and simple molecular assays that can reliably distinguish *M. leprae* from *M. lepromatosis* in routine diagnostics and in animal surveillance. In this study, we contribute to this effort by reporting the use of a highly repetitive genomic region, present in 38 copies, enabling sensitive and specific molecular detection of *M. lepromatosis*.

**FIGURE 1:**
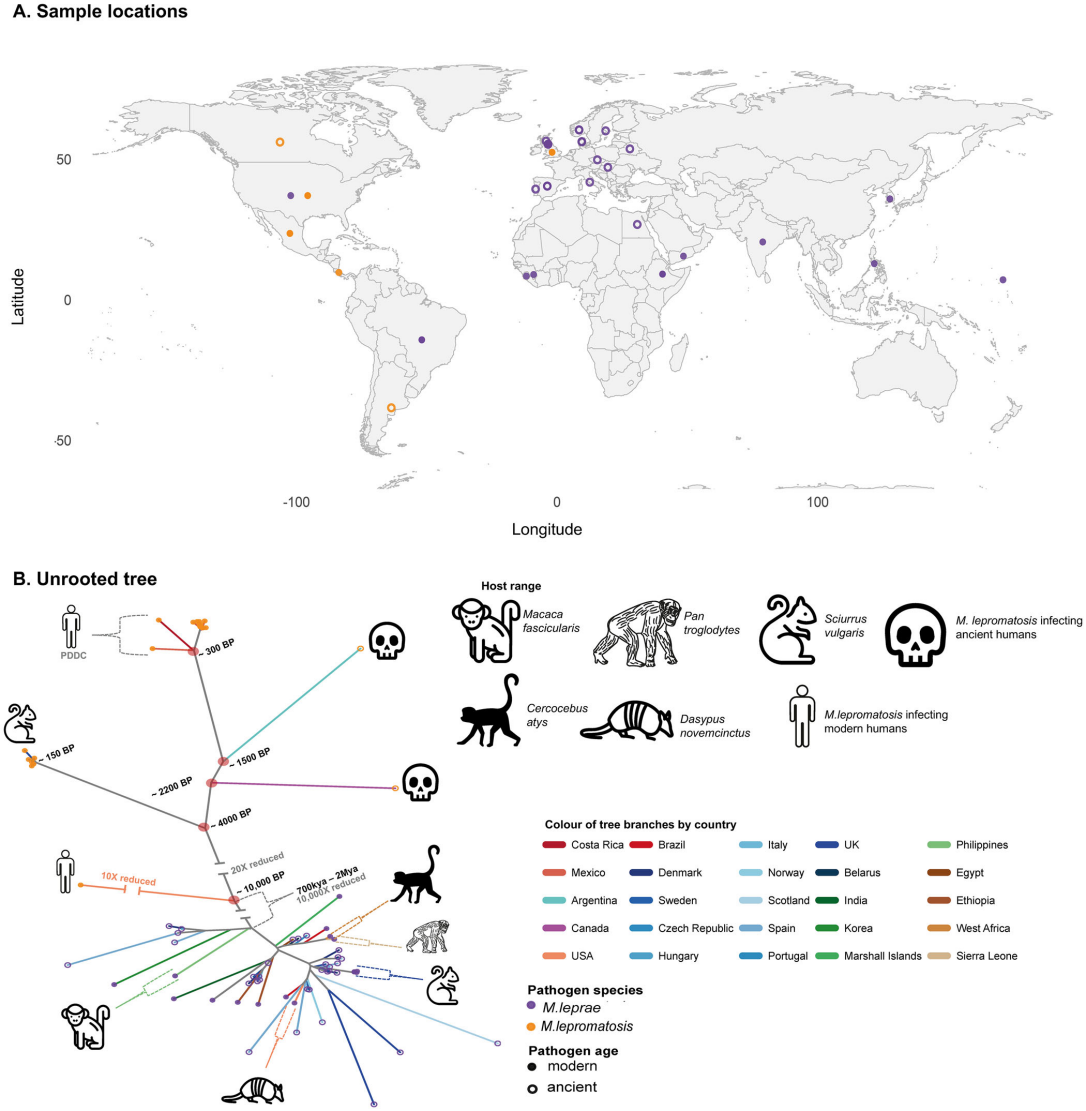
Geographic distribution and phylogenetic relationships of *Mycobacterium leprae* and *M. lepromatosis*. (A) Map showing the location of the 70 high‑quality ancient and modern genomes of *M. leprae* (purple) and *M. lepromatosis* (orange). Sampling locations are approximate and reflect general geographic origins rather than exact find sites. Ancient samples (open circles) overlap geographically with modern ones, indicating long‑term persistence. (B) An unrooted phylogenetic tree of the 70 leprosy bacilli, high‐quality ancient and modern genomes, including wild animal infecting strains. *M. lepromatosis* sequences form a well‑defined (tip shapes in orange), separate clade from *M. leprae* (tip shapes in purple), confirming their deep evolutionary divergence. For visualisation purposes, some branches have been rescaled: the divergence between the two species by 10,000X, the branch leading to NHDP‑LPM‑9 by 10X, and the branch between the modern squirrel British Isles strains and the deep divergent NHDP‑LPM‑9 lineage by 20X. Within *M. leprae*, country‑coloured branches show both regional clustering and intercontinental mixing, reflecting historical transmission routes. Animal‑infecting *M. leprae* strains are nested within the diversity observed among human isolates, in contrast to *M. lepromatosis*, for which red squirrel strains constitute a separate clade. These 70 high‑quality genomes capture the temporal and geographic breadth of leprosy pathogens. The tree illustrates a deep split between *M. leprae* and *M. lepromatosis*, and deep evolutionary histories in certain lineages of *M. lepromatosis*.

A broader epidemiological perspective emerges when we compare *M. lepromatosis* and *M. leprae* side by side. In phylogenetic trees including both species, *M. leprae* typically shows relatively short branches and dense clustering within lineages, consistent with a more recent and better sampled diversity, associated with human radiation and the rapid global spread that was facilitated by historical human mobility and trade networks,^8^ (Figure 1B). Animal‐infecting clades fall within this human‐related diversity^2^, ^9^, ^10^ contrasting with *M. lepromatosis*, in which an animal‐associated lineage forms a distinct, deeply diverging clade. By contrast, *M. lepromatosis* displays deeper splits and longer internal branches, consistent with older diversification and longer periods of geographic separation–patterns that are easier to reconcile with a multi‐host ecology and/or long‐term persistence in reservoirs that are incompletely sampled. These differences sharpen a key question: why did *M. leprae* become dominant in the Americas after European contact if *M. lepromatosis* was already endemic? Multiple nonexclusive mechanisms could contribute, including differences in transmissibility in dense or newly structured populations (such as those from colonial and post‐colonial times), differences in host adaptation, founder effects associated with repeated introductions, or ecological disruption during colonisation that altered exposure routes and reservoir dynamics. In this view, the post‐contact period does not represent the “arrival of leprosy” as a disease, but rather a major shift in the relative contribution of two causative agents, one long‐standing and likely ecologically complex (*M. lepromatosis*) and the other globally dispersed and, in principle, more tightly associated with human activity (*M. leprae*). Understanding this duality is not only historically important: it provides a framework for interpreting modern heterogeneity in case detection, zoonotic signals, emergence and re‐emergence sources and lineage‐specific dynamics across endemic regions.

Taken together, these results motivate a renewed biomedical and public health agenda. We now need to apply diagnostics that explicitly resolve closely related leprosy agents, genomic surveillance that spans endemic regions in the Americas and extends into Asia, and One Health investigations designed to identify animal reservoirs and transmission interfaces. In parallel, expanding comparative genomics and integrating protein‐structure and functional prediction may offer a practical route to prioritise candidate mutations that may underlie host shifts, immune evasion, or environmental adaptation. Clarifying how two deeply divergent pathogens have produced clinically overlapping disease is essential for understanding present‐day transmission, anticipating re‐emergence, and improving leprosy control in the decades ahead.

## AUTHOR CONTRIBUTIONS

*Conceptualization*: Maria Lopopolo and Nicolás Rascovan. *Writing*: Maria Lopopolo, Charlotte Avanzi and Nicolás Rascovan. *Figure*: Maria Lopopolo.

## CONFLICT OF INTEREST STATEMENT

The authors declare no conflict of interest.
